# Supplementary material for: Late-fall satellite-based soil moisture observations show clear connections to subsequent spring streamflow
Source: Nat Commun. 2023 Jun 15;14:3545. doi: 10.1038/s41467-023-39318-3 (PMC10272137; doi:10.1038/s41467-023-39318-3)
Supplement: Supplementary file 1 — Supplementary Information [file 41467_2023_39318_MOESM1_ESM.pdf]

Supplementary Material for

Late-Fall Satellite-based Soil Moisture Observations Show Clear Connections to Subsequent Spring Streamflow

Randal D. Koster[1*], Qing Liu[1,2], Wade T. Crow[3], and Rolf H. Reichle[1]

[1]Global Modeling and Assimilation Office, NASA Goddard Space Flight Center, Greenbelt, MD, USA

[2]Science Systems and Applications, Inc., Lanham, MD, USA

[3]U.S. Dept. of Agriculture, Agricultural Research Service, Hydrology and Remote Sensing Laboratory, Beltsville, MD, USA.

Corresponding author: Randal Koster (randal.d.koster@nasa.gov)

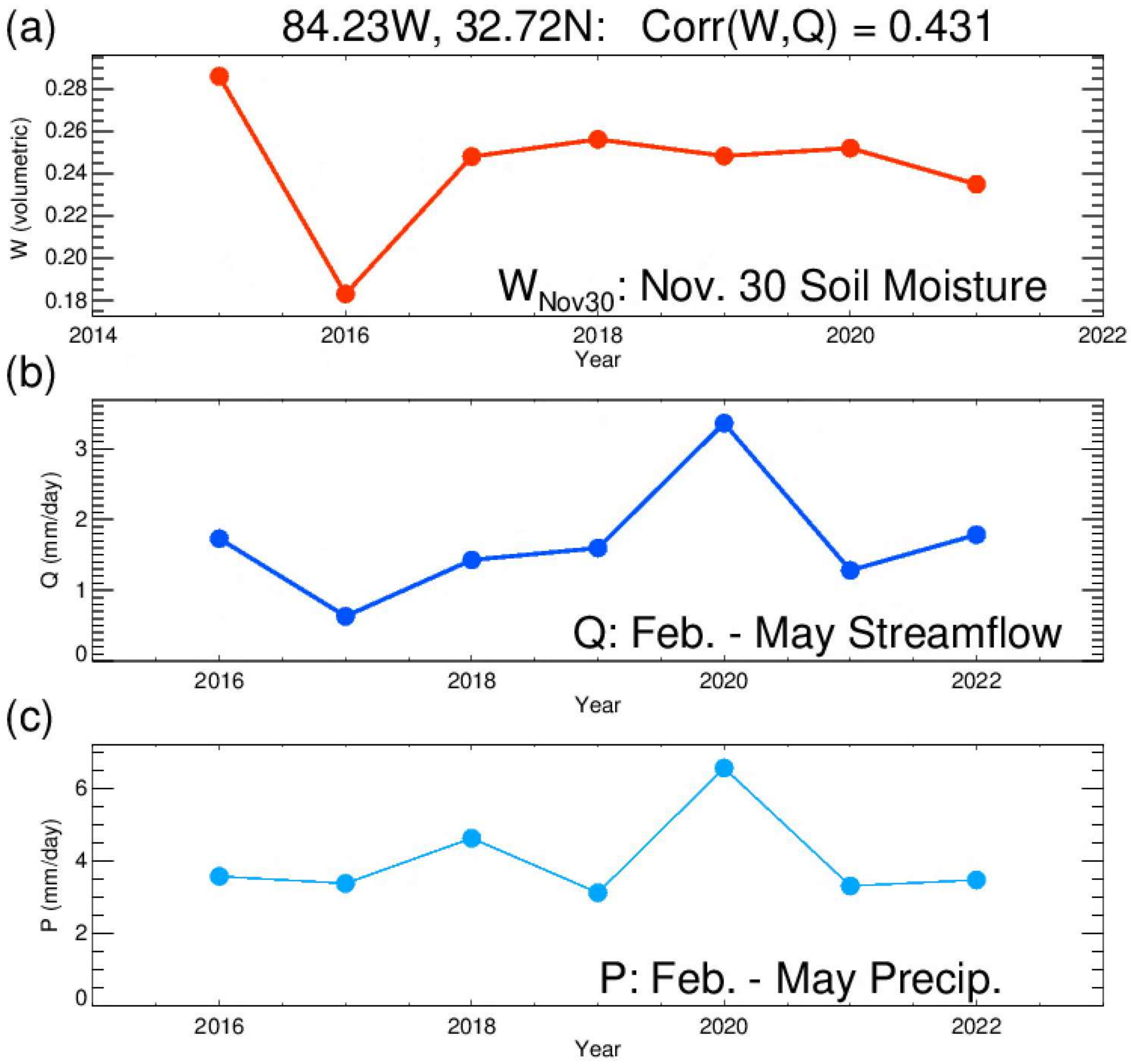

Figure S1. **Time series of hydrological quantities for the hydrological basin with the stream gauge measurement station at 84.23W, 32.72N**. In this basin, the correlation between November 30 profile soil moisture estimates (W) and total February–May streamflow (Q) is similar to the average found over CONUS. (a) yearly values of W (m^3 m^{-3}) as determined by applying the exponential filter to SMAP L2 soil moisture retrievals (see main text); (b) yearly values of measured Q for the basin (mm d^{-1}); and (c) yearly values of total February–May precipitation (P) measured for the basin (in mm d^{-1}, as extracted from the modeling system underlying the SMAP Level 4 product[1].) While W for 2019 does not predict the high Q in 2020 (which instead is clearly tied to high precipitation in 2020), the low W in 2016 does appear connected to the low Q for 2017.

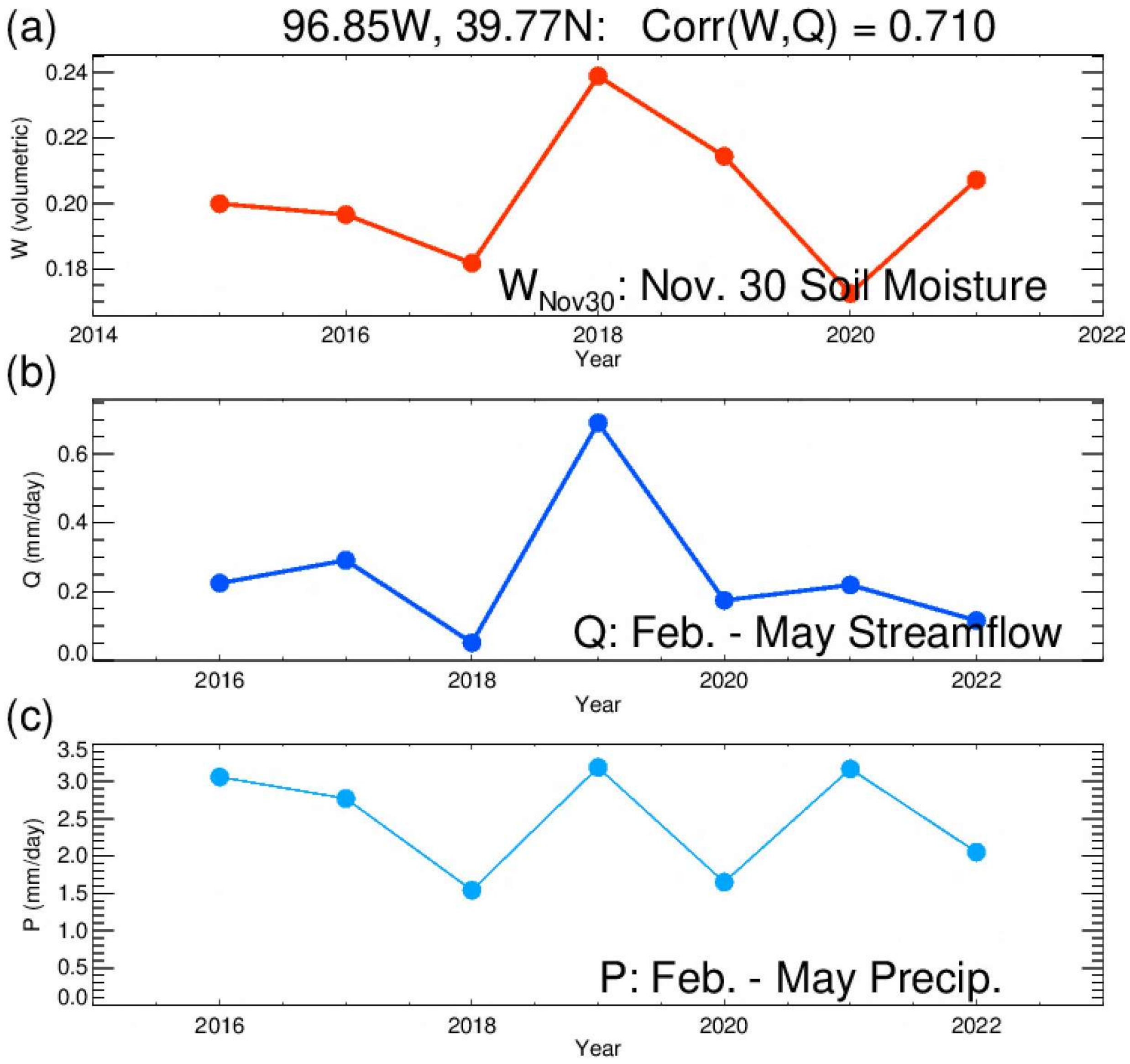

Figure S2. **As in Figure S1, but for the hydrological basin with the stream gauge measurement station at 96.85W, 39.77N.** In this basin, the correlation between W and Q is relatively high (0.71), in large part due to an apparent connection between high soil moisture in 2018 (top panel) and high subsequent streamflow in 2019 (middle panel).

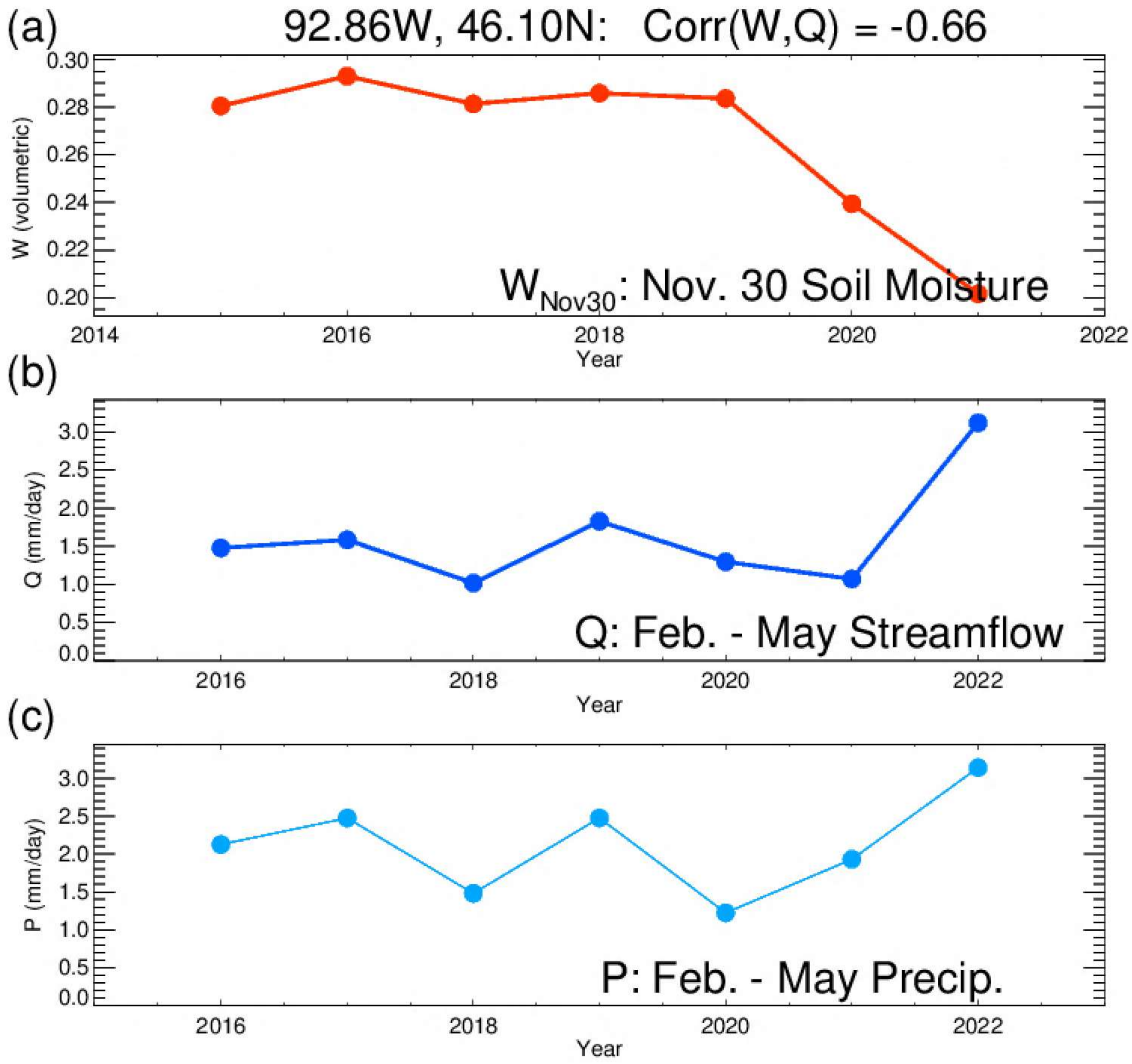

Figure S3. **As in Figure S1, but for the hydrological basin with the stream gauge measurement station at 92.86W, 46.10N.** This is one of the handful of basins for which the correlation between W and Q is negative; the correlation of -0.6 is indeed one of the largest negative values seen in the study. It largely results from the inability of the very low 2021 soil moisture value (top panel) to predict the very high 2022 streamflow value (middle panel), which itself was induced by high 2022 precipitation (bottom panel).

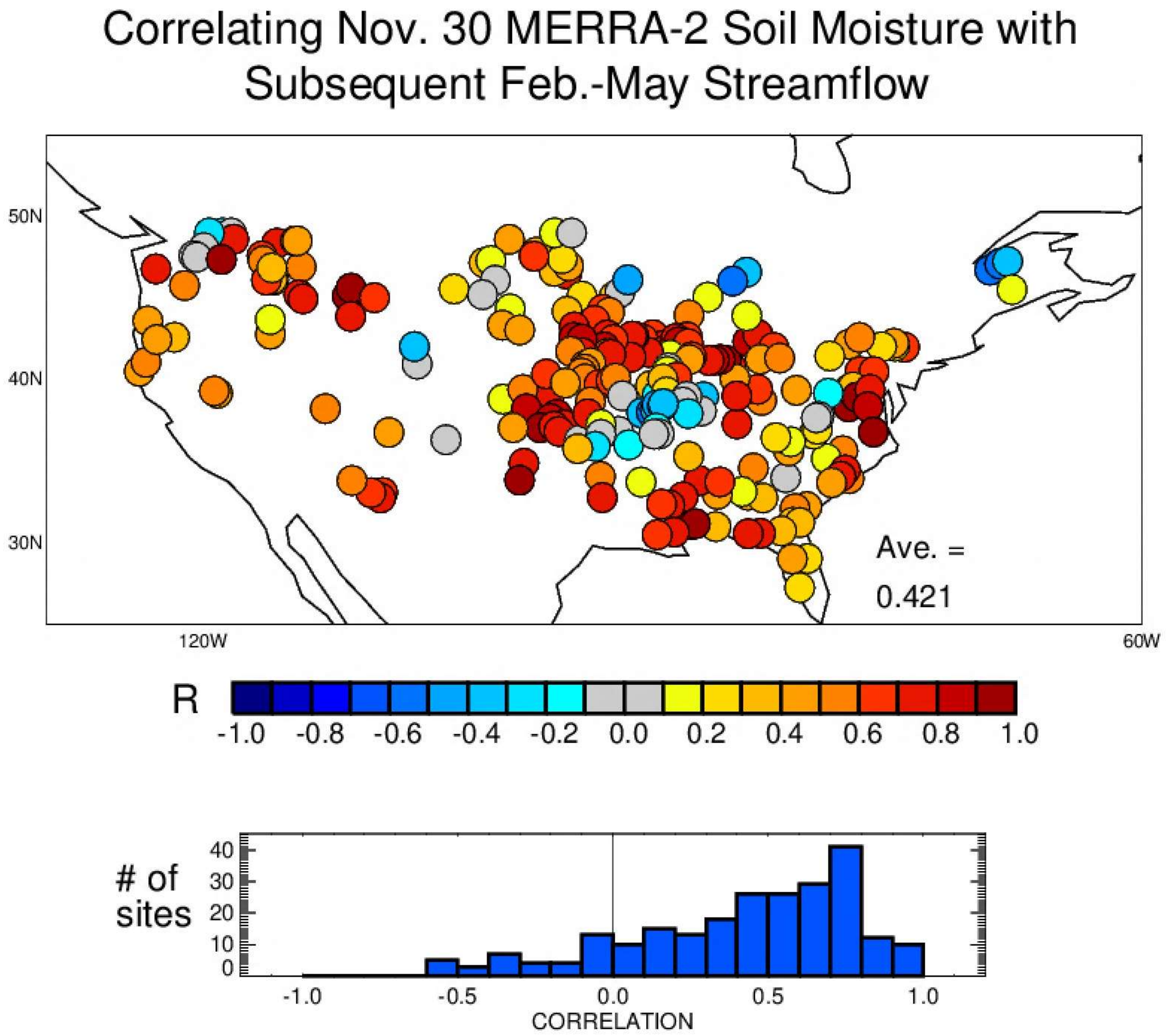

Figure S4: **Results using MERRA-2 profile soil moistures.** As in Figure 2a of the main text, but using November 30 MERRA-2 profile soil moistures as the predictor of springtime streamflow rather than the filtered SMAP data.

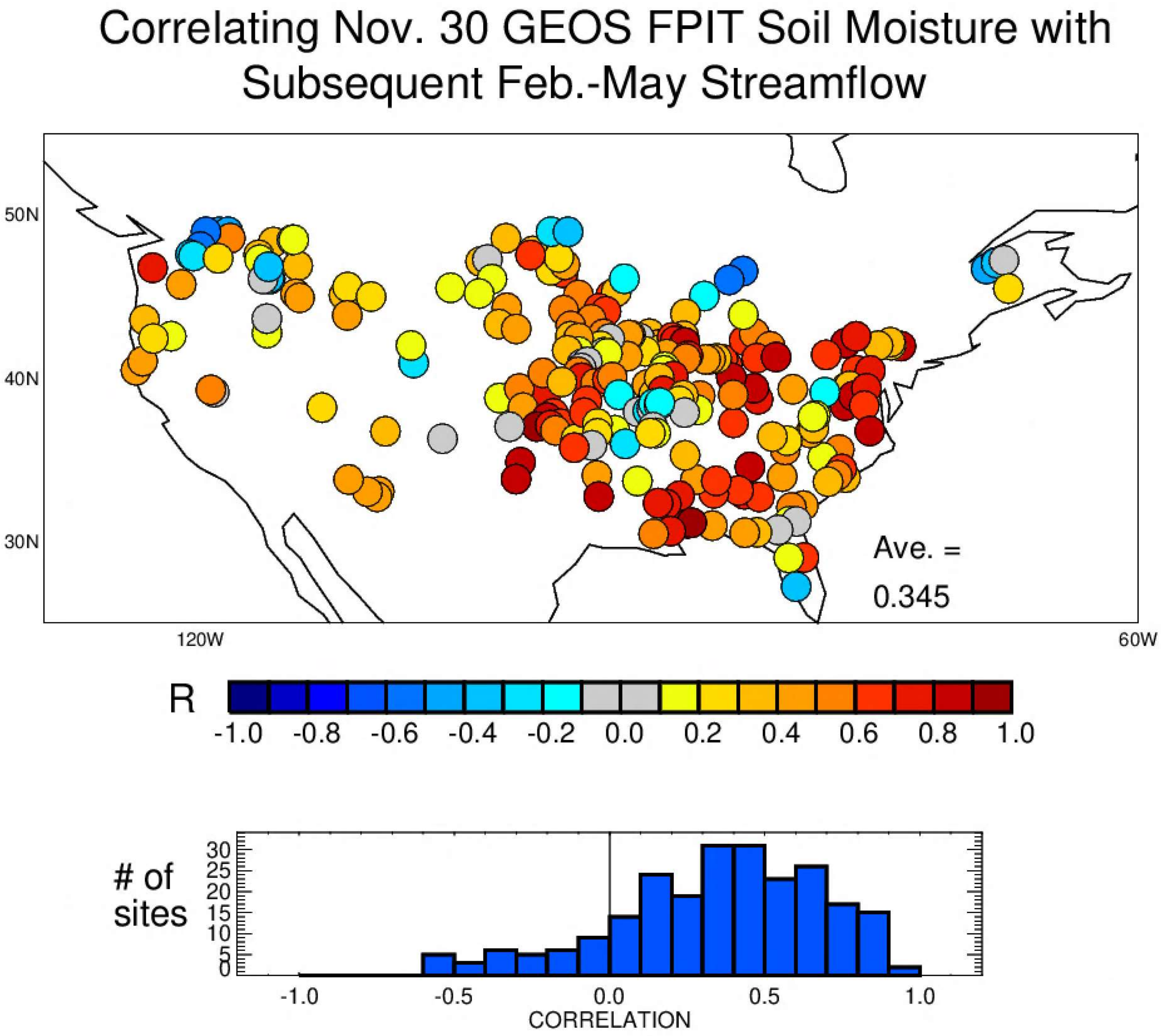

Figure S5: **Results using GEOS FPIT profile soil moistures.** As in Figure 2a of the main text, but using November 30 FPIT profile soil moistures as the predictor of springtime streamflow rather than the filtered SMAP data.

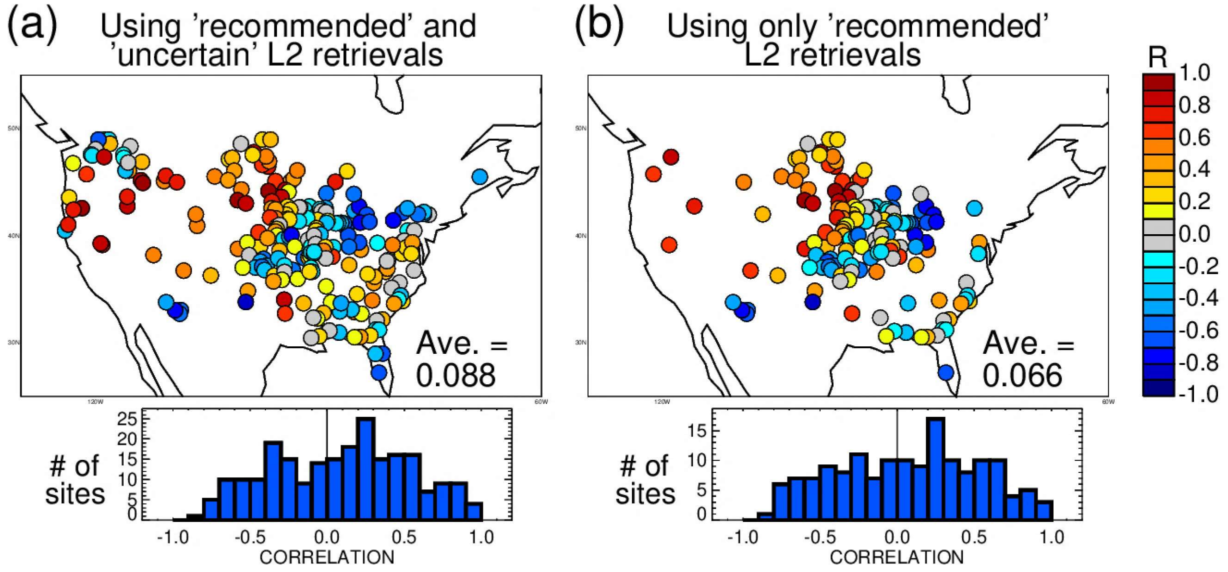

Figure S6: **Results for summertime SMAP-streamflow correlations.** As in Figure 2 of the main text, but for a lead that spans summer months: the correlations between April 30 profile soil moisture and streamflow averaged over July – October. Each correlation is based on 7 data pairs, with each pair extracted from a year within 2016-2022. The values of τ used to generate the soil moisture estimates underlying the calculations for the two panels are 31 and 47 days, respectively, as determined in summer-specific calibration exercises. While the correlations seem generally positive in the west and thus perhaps reflect some true predictability, the eastern half of the continent shows no preponderance of positive values, in clear contrast to Figure 2.

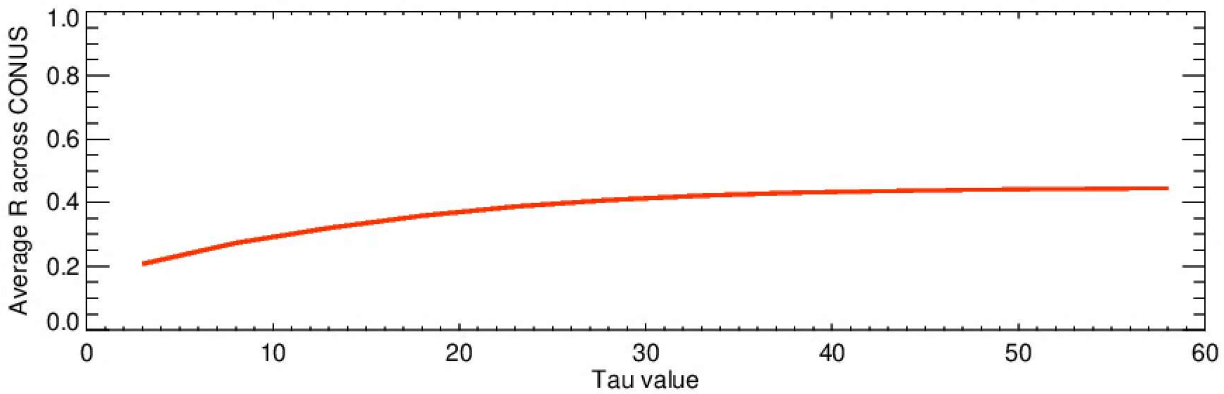

Figure S7: **SMAP-streamflow correlations as a function of filtering timescale.** Average correlation coefficient R, across the basins examined in CONUS, between the profile soil moisture estimates, W_{Nov30}, and subsequent spring streamflow assuming different values of the τ timescale parameter. Values of τ much smaller than that used in the main text (τ=38 days), which had been calibrated with independent data, would have produced significantly smaller average correlations.

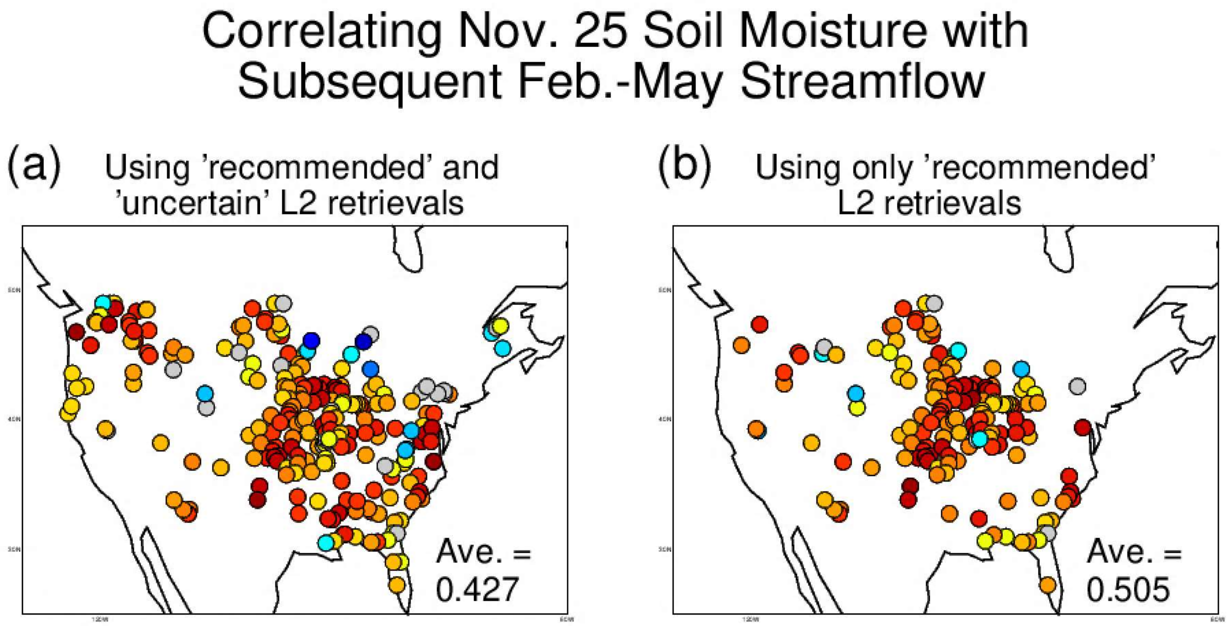

Figure S8. **Results for an earlier soil moisture predictor.** As in Figure 2 of the main text, but using exponentially filtered late-fall soil moisture for November 25 instead of November 30.

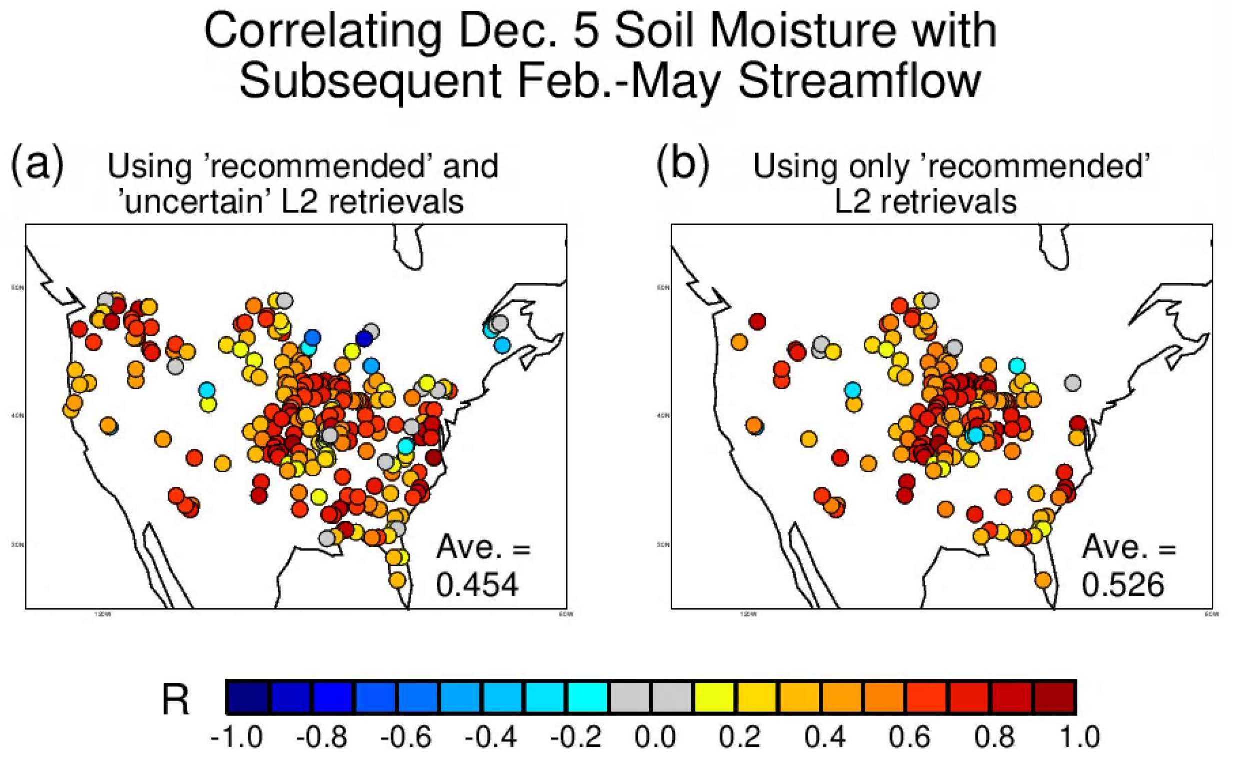

Figure S9. **Results for a later soil moisture predictor.** As in Figure 2 of the main text, but using exponentially filtered late-fall soil moisture for December 5 instead of November 30.

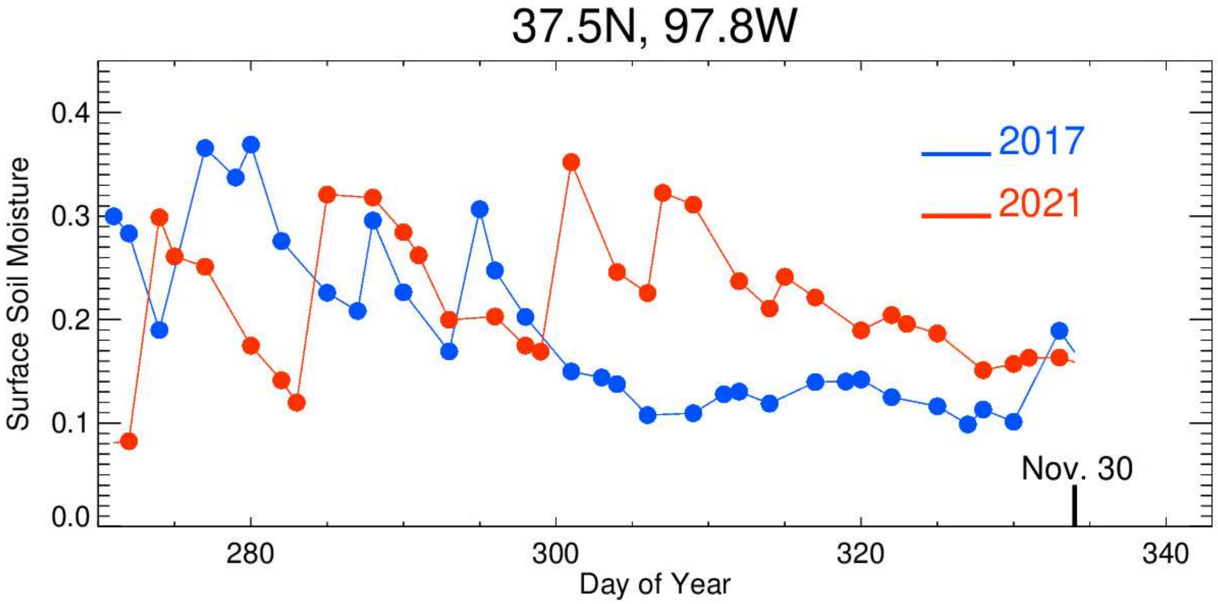

Figure S10. **Representative SMAP retrieval time series.** Fall time series of surface SMAP L2 retrievals over a representative EASE grid cell at 37.5 N, 97.8 W for two representative years. The SMAP radiometer measures conditions in the top several cm of soil, so that the individual retrievals (the dots in the figure) are subject to high frequency variability, responding quickly to rain events and to fast post-rain dry downs. Note, however, that on average after day 300, the soil moistures for 2021 lie well above those for 2017. By averaging the retrievals over time, we can remove the high frequency variability, leaving behind this low frequency signal. In this paper, we effectively assume that the low-frequency signal obtained by such averaging is a direct reflection of slow variations in the moisture of the deeper soil column[2,3]. That is, in this figure, we infer that W_{Nov30} in 2021 is wetter than that in 2017 even though 2017's surface soil moisture on November 30 is probably slightly wetter.

References

[1]Reichle, R. H., Liu, Q., Koster, R. D., Crow, W. T., De Lannoy, G. J. M., Kimball, J. S., et al. Version 4 of the SMAP Level-4 Soil Moisture Algorithm and Data Product. *Journal of Advances in Modeling Earth Systems*, **11**, 3106-3130 (2019).

[2]Albergel, C., Rudiger, C., Pellarin, T., Calvet, J.-C., Fritz, N., Froissard, R., et al. From near-surface to root-zone soil moisture using an exponential filter: an assessment of the method based on in-situ observations and model simulations. *Hydrol. Earth Syst. Sci.*, **12**, 1323-1337 (2008)

[3]Ford, T. W., Harris, E., & Quiring, S. M. Estimating root zone soil moisture using near-surface observations from SMOS. *Hydrol. Earth Syst. Sci.*, **18**, 139–154 (2014).
